# DNA Methylation of miR-122 Aggravates Oxidative Stress in Colitis Targeting SELENBP1 Partially by p65NF-*κ*B Signaling

**DOI:** 10.1155/2019/5294105

**Published:** 2019-03-24

**Authors:** Jianan Bai, Junchi Yu, Jintian Wang, Bingyan Xue, Na He, Ye Tian, Lixia Yang, Yipin Wang, Yanyan Wang, Qiyun Tang

**Affiliations:** ^1^The First Affiliated Hospital of Nanjing Medical University, Nanjing, China; ^2^The Third Affiliated Hospital of Nanjing University of Chinese Medicine, Nanjing, China; ^3^People's Hospital of Linyi County, Dezhou, China; ^4^The Affiliated Sir Run Run Hospital of Nanjing Medical University, Nanjing, China

## Abstract

Aberrant microRNA (miRNA) expressions contribute to the development and progression of various diseases, including Crohn's disease (CD). However, the accurate mechanisms of miRNAs in CD are definitely unclear. We employed colonic tissue samples from normal volunteers and CD patients, an acute mice colitis model induced by 2,4,6-trinitro-benzene-sulfonic acid (TNBS), and a cellular oxidative stress model induced by H_2_O_2_ in HT-29 cells to determine the effects of oxidative stress on expressions of miR-122, selenium-binding protein 1 (SELENBP1, SBP1), p65 nuclear factor *κ*B (p65NF-*κ*B) signaling, and DNA methylation. We found that SBP1 was mainly located on epithelial cells and was significantly increased in patients with active CD. SBP1 was the target gene of miR-122. miR-122 expression was downregulated while SBP1 expression was upregulated under TNBS-induced colitis or oxidative stress. Pre-miR-122 or siRNA SBP1 (si-SBP1) treatment ameliorated acute TNBS-induced colitis and H_2_O_2_-induced oxidative stress. Cotreatment of pre-miR-122 and si-SBP1 enhanced these effects. Besides, pre-miR-122 and si-SBP1 obviously activated the p65NF-*κ*B signaling by phosphorylation of I*κ*B*α*. Bisulfite sequencing of the CpG islands in the promoter region of miR-122 showed that CpG methylation was significantly increased under oxidative stress. Treating cells with 5′-AZA which was well known as a DNA-demethylating agent significantly increased miR-122 expression. Our results suggest that oxidative stress-induced DNA methylation of miR-122 aggravates colitis targeting SELENBP1 partially by p65NF-*κ*B signaling and may promote the progression of CD.

## 1. Introduction

Crohn's disease (CD), which is involved in inflammatory bowel disease (IBD), is a chronic refractory gastrointestinal colitis. It usually appears on juvenile and brings global burden to all over the world [1]. The annual incidence of CD soars to about 12.7/100,000 in Europe and 5/100,000 in Asia [2, 3]. However, although there are some effective treatments on CD at present, patients still need long-term medication and even undergo surgery [4, 5]. To some extent, it is a huge challenge to explore the pathogenesis and develop new therapeutic targets of CD.

Oxidative stress has been found to play a key role in the pathogenesis and progression of CD [6]. In a normal intestinal mucosa, there is a balance between oxidative stress factors such as reactive oxygen species (ROS), hydrogen peroxide (H_2_O_2_), superoxide anion (O^2-^), and antioxidant molecules such as glutathione (GSH), superoxide dismutase (SOD), and glutathione-S-transferase (GST) [7–9]. In the intestinal mucosa of CD, infiltrating inflammatory cells release excessive ROS, while SOD and other antioxidant enzymes decrease, leading to oxidative stress enhancement, lipid peroxidation, which ultimately causes intestinal mucosal damage [10, 11]. Enhanced oxidative stress can result in serious cell damage directly as well as activating nuclear factor *κ*B (NF-*κ*B) signaling and the following proinflammatory factors (IL-6, IL-8, etc.) and intracellular adhesion molecules (ICAM) [12, 13].

Selenium-binding protein 1 (SELENBP1, SBP1), a member of the selenoprotein family, widely distributes in a variety of tissues, with a high level in the heart and a moderate level in the liver, lung, kidney, and intestinal tissues [14]. A few studies have reported the anticancer effect of SBP1 in renal cell carcinoma, head and neck cancers, and prostate cancer [15–17]. However, the role of SBP1 in oxidative stress and CD remains largely unknown.

To our knowledge, microRNAs (miRNAs) are a series of endogenous small molecules, regulating the pathogenesis of many diseases, such as inflammation, autoimmune diseases, cancer, and CD [18–21]. The mature miRNAs bind to the target mRNA through RNA-induced silencing complex (RISC) and complement to the 3′-untranslated region of target mRNA, causing degradation or translation inhibition of target mRNA [22]. A number of abnormal expressed miRNAs have been selected in CD, which not only participate in the pathogenesis of CD but also attract more and more attention in the progression of the diagnostic and therapeutic potentials [21, 23]. miR-122, known as a liver-specific miRNA, involved in the pathology of liver diseases, such as hepatitis and liver cancer [24, 25]. Antisense oligonucleotide miravirsen targeting miR-122, as the first microRNA-targeted drug, has been used in clinical trials for the treatment of hepatitis C virus (HCV) infection [26].

Until now, we have confirmed that miR-122 is closely related to oxidative stress in a rat liver injury model [27]. However, whether miR-122 mediates oxidative stress in CD and its mechanisms has not been reported. Therefore, this study was designed to investigate the potential role of miR-122 on oxidative stress and inflammation in CD, which would provide a theoretical basis for an effective target of diagnosis and treatment of CD.

## 2. Materials and Methods

### 2.1. Mucosal Biopsy Specimens

Biopsy tissues were obtained from inflamed and noninflamed mucosa of patients with active CD (*n* = 6) who were diagnosed according to clinical and macroscopic criteria. Control samples were collected from healthy volunteers (*n* = 6). They were fixed in formalin and embedded in paraffin for an immunohistochemistry assay.

### 2.2. Establishment of an Acute CD Model and Assessment

Female Balb/c mice aged 7 weeks were purchased from the Laboratory Animal Center of Nanjing Medical University (Nanjing, China). The acute CD model was induced with TNBS (Sigma, USA) as published previously [28]. Briefly, 3 mg of TNBS in 100 *μ*L of 50% ethanol was slowly administered via a transrectal polyethylene catheter inserted 4 cm from the anus (TNBS, *n* = 20). An equivalent volume of 50% ethanol was instilled into the control group (ethanol, *n* = 20).

Mice were killed by cervical dislocation on days 1, 3, 5, 7, and 10 to get the most severe one. To determine the effect of pre-miR-122 in CD, mice were treated with pre-miR-122 or siRNA (5 mg/kg) for 12 h after TNBS injection and killed on day 3. To evaluate the degree of colitis, the disease activity index [29] was calculated involving body weight (ranged from 0 to 4), fecal consistency (ranged from 0 to 4), and the presence of a bloody stool (ranged from 0 to 4) daily after TNBS treatment. Meanwhile, hematoxylin and eosin staining on the lowest 4 cm colon was performed, and the histology score was calculated involving edema (ranged from 0 to 3) and depth of inflammatory cell infiltration (ranged from 0 to 3) on the bowel wall.

### 2.3. Cell Culture

The human intestinal epithelial HT-29 cell line was purchased from Chinese Academy of Sciences (Shanghai, China) and cultured in Dulbecco's modified Eagle's medium (DMEM; Gibco, CA, USA) supplemented with 10% fetal bovine serum (FBS, Gibco), 100 U/mL penicillin, and 100 *μ*g/mL streptomycin (Gibco, CA, USA).

### 2.4. Ectopic Expression of miR-122

Synthetic miR-122 precursor (pre-miR-122, GenePharma, Shanghai, China) and scrambled negative control RNA (NC) were added to HT-29 cells with Lipofectamine 2000 (Invitrogen, USA) when cells were approximately 70% confluent. Twenty-four hours after transfection, the cells were used for the special experiments.

### 2.5. SBP1 Inhibition

Specific SBP1 siRNA (si-SBP1, GenePharma, Shanghai, China) and negative control siRNA (si-NC) were transfected, respectively, when cells were approximately 50% confluent. Lipofectamine 2000 (Invitrogen, USA) and siRNA (80 nM/L) were used in a total serum-free medium of 2 mL per well. After 8 h, 2 mL of culture medium was added to each well for another 40 h.

### 2.6. Cell Viability Assay

Cell viability was evaluated by Cell Counting Kit-8 (CCK-8, Dojindo, Japan) following the manufacturer's instructions. Twenty-four hours after treatment with H_2_O_2_ (0, 200, 400, 600, 800, and 1000 *μ*M), approximately 2 × 10^3^ cells were seeded into each well of 96-well plates. The value of each sample was measured on a microplate reader (Bio-Rad, USA) at three different time points.

### 2.7. Cytokine Enzyme-Linked Immunosorbent Assays

HT-29 cells were then stimulated with H_2_O_2_ (0, 100, 200, 300, 400, 500, and 1000 *μ*M) for 24 h or left untreated. The levels of MDA, SOD, and ROS were measured in culture supernatants using enzyme-linked immunosorbent assay (ELISA) kits (R&D Systems, Minneapolis, MN, USA). Absorbance was measured at 450 nm and compared with the respective standard curve of the cytokines.

HT-29 cells were pretreated with pre-miR-122, si-NC, si-SBP1, and both pre-miR-122 and si-SBP1 for 24 h. Pretreated cells were then stimulated with H_2_O_2_ (300 *μ*M) for another 24 h or left untreated. The related levels of ROS, MDA, 8-OHdG, IL-6, and IL-8 were measured in culture supernatants.

As to the expressions of 8-OHdG, GSH, MDA, ROS, IL-6, and IL-8 in the TNBS-induced colitis model, full-thickness colonic tissues of mice were homogenized in protease inhibitors (KeyGen Biotech, Nanjing, China) supplemented with 1 mM phenylmethane sulphonyl fluoride (PMSF) to obtain the supernatants, which were finally tested for protein level. To measure these cytokines, mouse double-antibody sandwich ELISA kits (R&D Systems) were developed.

### 2.8. RNA Isolation and qRT-PCR

Total RNA was isolated and extracted with the miRNeasy Mini Kit (Qiagen, Germany) as the manufacturer's instructions. The complementary DNA (cDNA) was prepared from total RNA using the Omniscript RT kit (Qiagen, Germany). The levels of miR-122 and SBP1 were conducted with the miScript SYBR Green PCR kit (Qiagen, Germany) on a 7500 Real-Time PCR system (Applied Biosystems, USA). U6 and GAPDH were normalized for the expressions of miR-122 and SBP1, respectively. Primer pairs used in this study were

- miR-122: forward: 5′-TTGAATTCTAACACCTTCGTGGCTACAGAG-3; reverse: 5′-TTAGATCTCATTTATCGAGGGAAGGATTG-3′
- U6: forward: 5′-CTCGC TTCGGCAGCACA-3′; reverse: 5′-AACGCTTCACGAATTTGCGT-3′
- SBP1: forward: 5′-CCCATTGCTTCCACAGCTACGA-3′; reverse: 5′-GCCCTTCACTTTCTTGGGGG-3′
- GAPDH: forward: 5′-GAGTCAACGGATTTGGTCGT-3′; reverse: 5′-TGTGGTCATGAGTCCTTCCA-3′

The 2^-ΔΔCt^ method was used to evaluate the relative miRNA expression levels for cells and tissue samples.

### 2.9. Protein Extraction and Western Blotting

Total cell lysates or tissue homogenates were prepared by using a RIPA lysis buffer (Beyotime, China). Conventional Western blotting experiments were performed. SBP1, p-p65, p65NF-*κ*B, p-I*κ*B*α* primary antibodies (Abcam, MA, USA), ICAM, and GAPDH (Santa Cruz, CA, USA) primary antibodies were incubated overnight at 4°C. The protein levels were normalized to GAPDH. Alpha Innotech (San Leandro, CA) imaging software was used to quantify these data.

### 2.10. Luciferase Reporter Assay

SBP1 3′-untranslated region (3′-UTR) was mutated using the mutagenesis kit (Promega, USA). Wild-type and mutant sequences were amplified and inserted into the vector to construct luciferase reporter plasmids following the manufacturer's instructions (Promega, USA). Cells were seeded in 96-well plates and cotransfected with reporter constructions (Wt vector and Mut vector) and miR-122. The luciferase activity was measured using the dual-luciferase reporter assay (Promega, USA).

### 2.11. Methylation-Specific PCR and Sequencing

HT-29 cells were pretreated or non-pretreated with DNA-demethylating agent 5′-AZA-2′-deo-xycytidine (5′-AZA, 1 *μ*M, Sigma-Aldrich, USA) and incubated under normal or H_2_O_2_. Genomic DNA was isolated by standard phenol chloroform extraction and ethanol precipitation using a commercially available DNA extraction Kit (Magen, China). The DNA was modified with sodium bisulfite using the EpiTect TM Bisulfite Kit (Qiagen, Hilden, Germany) according to the manufacturer's protocol.

To amplify the CpG islands in the promoter region of the miR-122 gene, the sequence was amplified using the primers (F) ATTTTAATTTATGGGAGTAGAACGA and (R) ATCTTACTTTAACCCTAAAACCGAC. The bisulfite-treated PCR products were purified with a commercially available extraction kit (Magen, China) and cloned into the pMD-18T vector (TaKaRa, Japan). Ten clones from each sample were sequenced.

### 2.12. Statistical Analysis

GraphPad Prism 5 software (GraphPad Software, San Diego, CA) was performed to carry out all statistical analyses. One-way analysis of variance was used for multiple group comparison. When only two groups were compared, Student's *t*-test was performed. *p* values of less than 0.05 (*p* < 0.05) were considered to be significant. All quantitative data were expressed as the mean ± SEM.

### 2.13. Ethical Considerations

All animals received appropriate care according to the requirements of the Animal Care and Use Committee of Nanjing Medical University. All patients and normal control individuals signed a written informed consent form.

## 3. Results

### 3.1. SBP1 Expression Was Increased in Patients with Active CD

**Figure 1:**
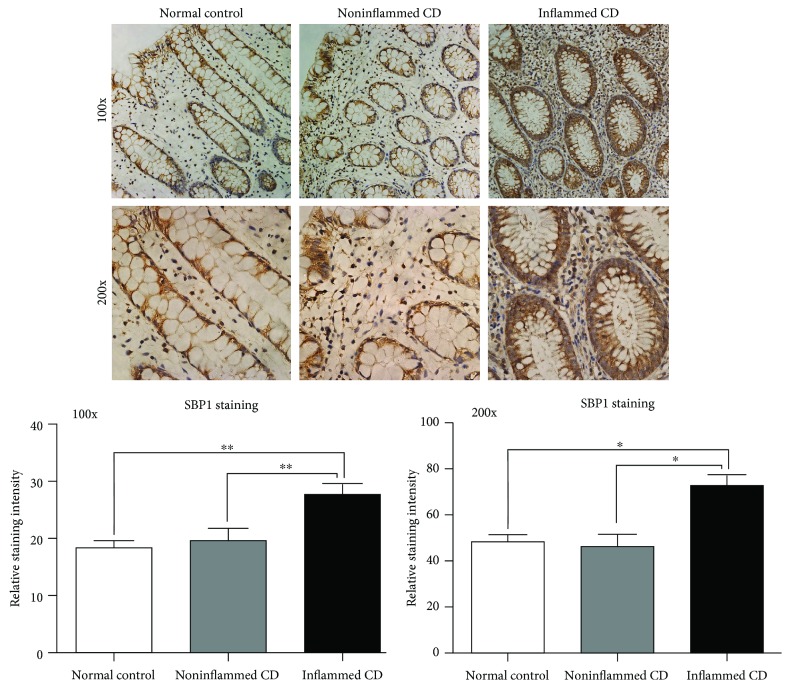
SBP1 expression was increased in patients with active CD with immunohistochemistry staining. Weak staining of SGK1 was detected in the samples from normal controls, while strong staining of SGK1 was observed in samples from patients with active CD.

Strong SBP1 staining was observed in the samples from patients with active CD while relatively weak SBP1 staining was observed in the normal group. SBP1 mainly located in the cytoplasm of IECs (Figure 1).

### 3.2. TNBS Induced Severe Colitis and SBP1 Was Increased in This Colitis

**Figure 2:**
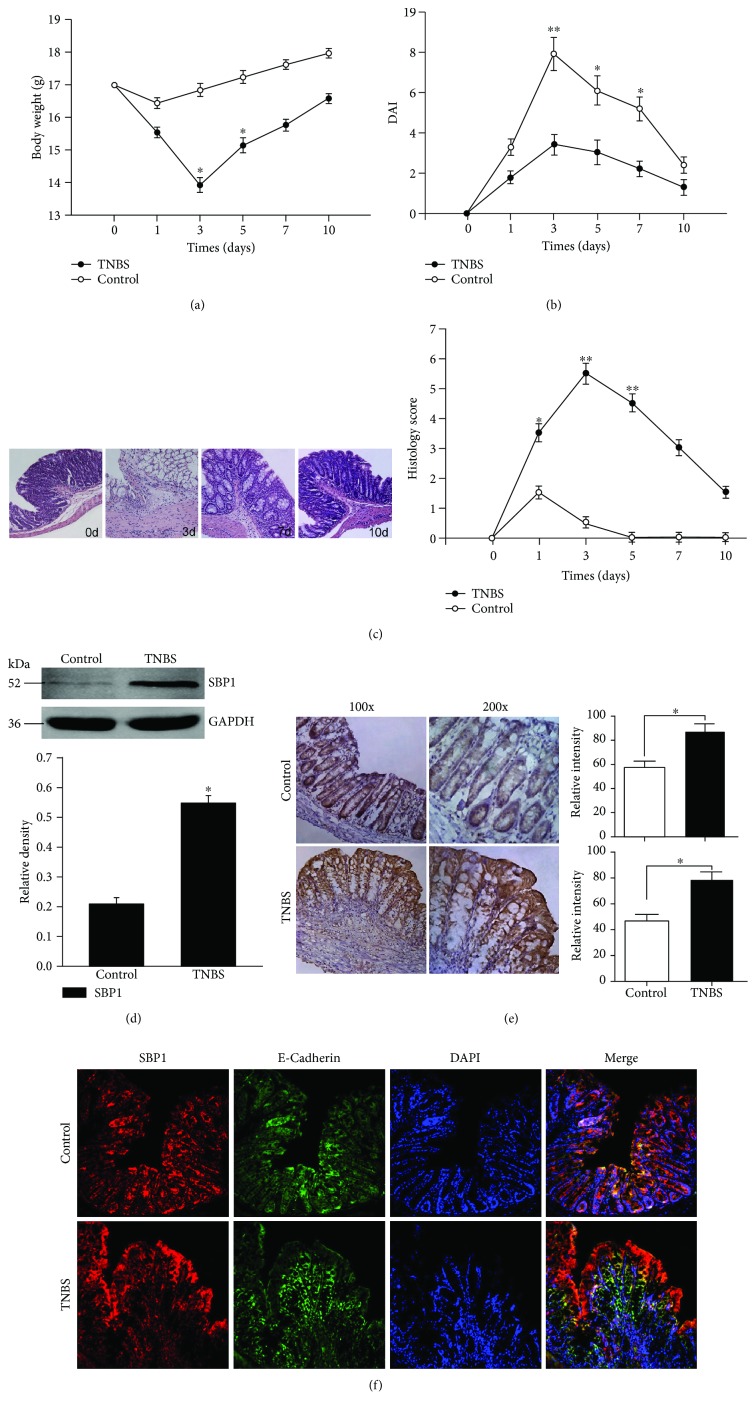
High expression of SBP1 in the established TNBS-induced colitis model. (a–c) TNBS or ethanol (control group) administration caused variation about body weight, DAI, and histology score in the murine model. (d, e) Western blotting analysis and immunohistochemical staining were performed to study the SBP1 level in TNBS-induced colitis compared with ethanol-treated mice. SGK1-dependent staining is indicated by brown areas. (f) Colocalization of SBP1 and E-cadherin by the immunofluorescence assay indicated the localization of SBP1.

The TNBS-induced colitis group showed more obvious mean body weight loss compared with controls (50% ethanol). The mean body weight loss was 8.2% over 24 h and reached a peak of 18.8% at 72 h (Figure 2(a)). The mean DAI was 7.9 in the TNBS group while it was 3.67 in the control group at 72 h (Figure 2(b)). The pathology assay also showed the most severe colitis at day 3 (Figure 2(c)). The SBP1 level was higher in the TNBS group compared with the control group via the Western blotting assay (Figure 2(d)) and immunohistochemical staining (Figure 2(e)). SBP1 shared the same location with E-cadherin which indicated its expression on epithelial cells (Figure 2(f)).

### 3.3. Oxidative Stress Affected miR-122 and SBP1 Levels

**Figure 3:**
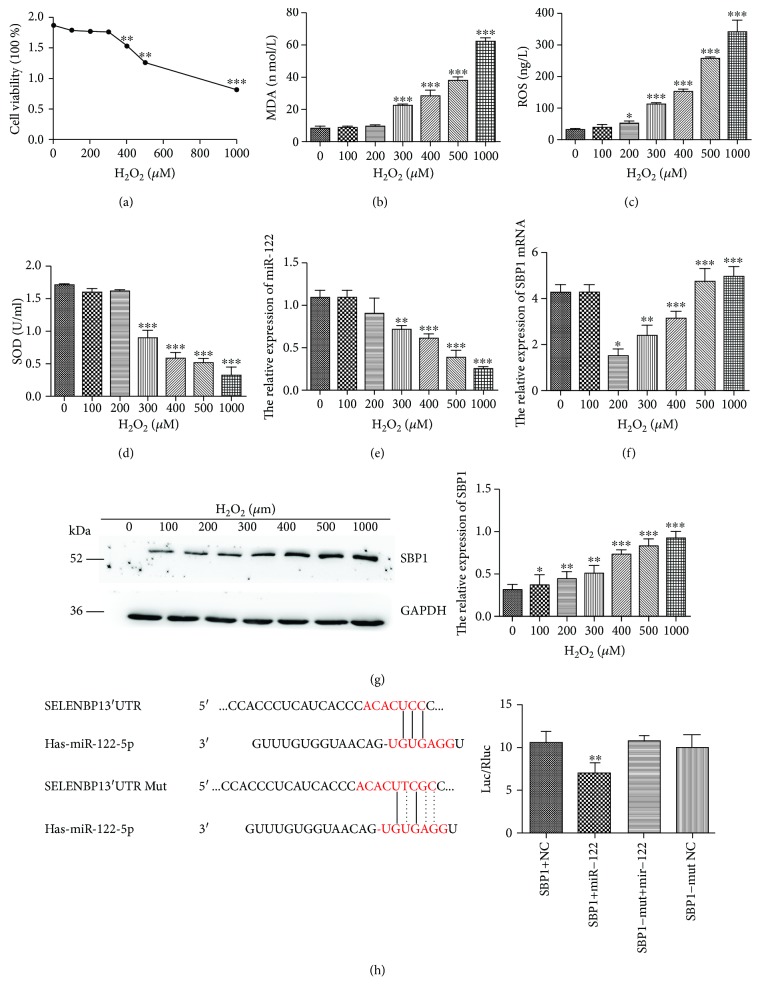
miR-122 was downregulated in the oxidative stress model of human intestinal epithelial cell line HT-29. (a) CCK-8 analysis of cell viability after treatment of H_2_O_2_ with different concentrations. (b–d) The protein levels of MDA, SOD, and ROS in the supernatant of culture medium under different concentrations of H_2_O_2_ were examined by ELISA. (e, f) The relative levels of miR-122 and SBP1 mRNA in HT-29 cells were detected by qRT-PCR under different concentrations of H_2_O_2_. (g) Western blotting analysis of SBP1 expression in HT-29 cells with different concentrations of H_2_O_2_. (h) The prediction of the binding between miR-122 and SBP1 by TargetScan. The dual-luciferase reporter assay was performed to verify the interaction of miR-122 and SBP1. ^∗^*p* < 0.05, ^∗∗^*p* < 0.01, and ^∗∗∗^*p* < 0.001 vs. 0 *μ*M.

As demonstrated by CCK-8 viability assays, the HT-29 cell viabilities were markedly declined in the H_2_O_2_ group (400, 500, and 1000 *μ*M but not 100, 200, and 300 *μ*M) compared to the control group (Figure 3(a)). HT-29 cells administrated with H_2_O_2_ (300, 400, 500, and 1000 *μ*M) succeeded in a remarkably higher level of MDA and ROS accompanied by a decreased level of SOD with the ELISA assay (Figures 3(b)–3(d)). Thus, we selected H_2_O_2_ (300 *μ*M) for the following experiments because it could activate oxidative stress but not affect the viability of HT-29 cells at this concentration. Besides, we also found that HT-29 cells succeeded in the lower level of miR-122 expression and the higher level of SBP1 mRNA in the H_2_O_2_ group than the control group (Figures 3(e) and 3(f)). We next determined SBP1 expression after H_2_O_2_ treatment by Western blotting and found that the H_2_O_2_-induced SBP1 expression occurred in a dose-dependent manner and showed positive correlation with concentration of H_2_O_2_ (Figure 3(g)).

### 3.4. SBP1 Was Verified as a Target of miR-122

**Figure 4:**
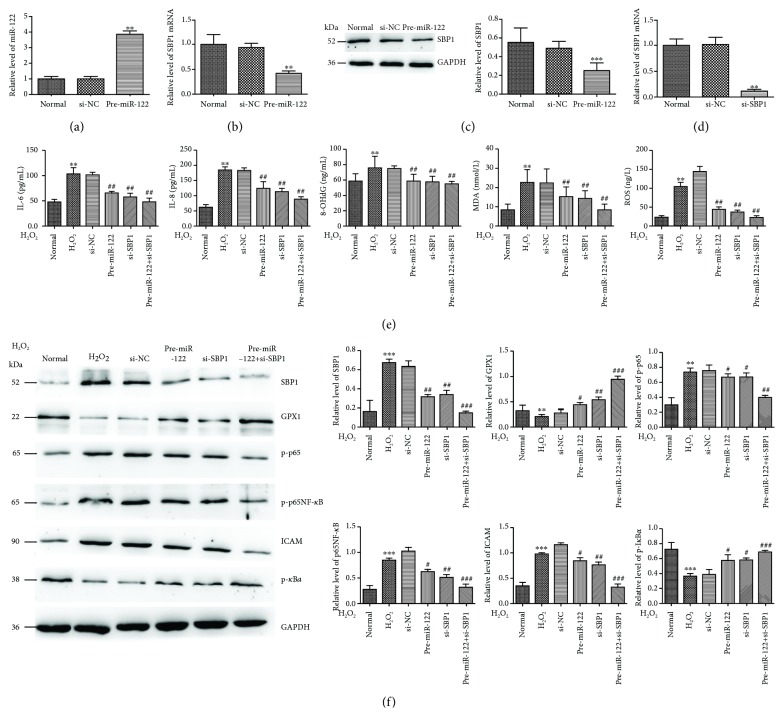
miR-122 protected H_2_O_2_-induced oxidative stress partially by p65NF-*κ*B signaling *in vitro*. (a–c) miR-122, SBP1 mRNA, and protein level in HT-29 cells after transfection with pre-miR-122. ^∗∗^*p* < 0.01 vs. si-NC. (d) The efficiency of si-SBP1. ^∗∗^*p* < 0.01 vs. si-NC. (e) The protein levels of ROS, MDA, 8-OHdG, IL-6, and IL-8 were examined by ELISA in normal, H_2_O_2_, pre-miR-122, si-SBP1, and both pre-miR-122 and si-SBP1-added H_2_O_2_ group. ^∗∗^*p* < 0.01 vs. normal; ^##^*p* < 0.01 vs. si-NC. (f) Lower levels of p-p65, p65NF-*κ*B, and ICAM and higher levels of GPX1 and p-I*κ*B*α* were found in the pre-miR-122 or si-SBP1-added H_2_O_2_ group than the H_2_O_2_ group. Meanwhile, cotreatment of pre-miR-122 and si-SBP1 could intensify these effects. ^∗∗^*p* < 0.01, ^∗∗∗^*p* < 0.001 vs. normal; ^#^*p* < 0.05, ^##^*p* < 0.01, ^###^*p* < 0.001 vs. si-NC.

To verify SBP1 as a target of miR-122, we searched the candidate target of miR-122 using the available database (TargetScan, miRanda, and PicTar) and found that 3′-UTR of SBP1 mRNA contained a high conserved binging site for miR-122. As shown in Figure 3(h), the luciferase activity was decreased in cells cotransfected with pre-miR-122 and 3′-UTR of SBP1 compared with the control group. However, no significant variation in luciferase activity was observed in cells with 3′-UTR-MUT of SBP1 with pre-miR-122 cotransfection. Then, we transfected pre-miR-122 into HT-29 cells to evaluate its efficiency and found significant increased expression of miR-122 compared to the control group (Figure 4(a)) followed by obviously decreased expression of SBP1 mRNA and protein (Figures 4(b) and 4(c)). These results suggested that SBP1 might be a direct target for miR-122. Meanwhile, we transfected si-SBP1 into HT-29 cells to evaluate its efficiency and found significant decreased expression of SBP1 compared to the control group (Figure 4(d)).

### 3.5. Pre-miR-122 or/and si-SBP1 Protected H_2_O_2_-Induced Oxidative Stress *In Vitro*

Then, cells pretransfected with pre-miR-122, si-SBP1, and both pre-miR-122 and si-SBP1 were then stimulated with H_2_O_2_ (300 *μ*M) for the indicated times. Subsequently, we found a higher level of ROS, MDA, 8-OHdG, IL-6, and IL-8 in the H_2_O_2_ group than the normal group, while a lower level of them in the pre-miR-122 and si-SBP1 group than the H_2_O_2_ group. Cotreatment of pre-miR-122 and si-SBP1 could intensify this phenomenon (Figure 4(e)).

Then, we examined the levels of ICAM and p65NF-*κ*B signaling related to oxidative stress and found significant activation of p65NF-*κ*B signaling and ICAM under H_2_O_2_ stimulation and pre-miR-122 or si-SBP1 treatment, respectively. However, we found lower levels of p-p65, p65, and ICAM and higher levels of GPX1 and p-I*κ*B*α* in the pre-miR-122 or si-SBP1-added H_2_O_2_ group than the H_2_O_2_ group. Meanwhile, cotreatment of pre-miR-122 and si-SBP1 could intensify these effects (Figure 4(f)).

### 3.6. Pre-miR-122 or si-SBP1 Treatment Ameliorated TNBS-Induced Colitis *In Vivo*

**Figure 5:**
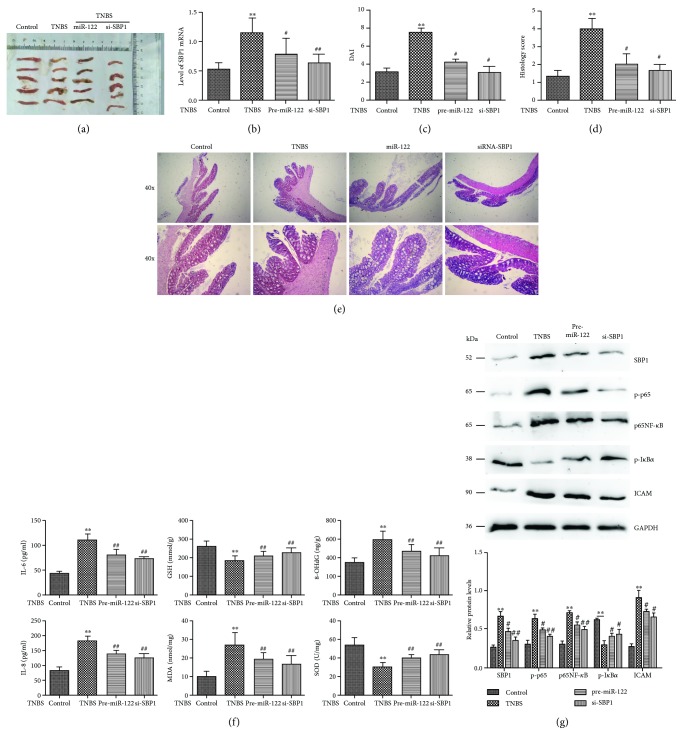
Pre-miR-122 or si-SBP1 treatment ameliorated TNBS-induced CD partially by p65NF-*κ*B signaling. (a) The macroscopic appearance of the inflammatory colon in the control, TNBS, pre-miR-122-added TNBS, and si-SBP1-added TNBS groups. ^∗∗^*p* < 0.01 vs. control (ethanol); ^#^*p* < 0.05, ^##^*p* < 0.01 vs. TNBS. (b) qRT-PCR analysis of SBP1 in the above groups. (c–e) DAI and histology scores in the above groups. ^∗∗^*p* < 0.01 vs. control; ^#^*p* < 0.05 vs. TNBS. (f) The protein levels of 8-OHdG, GSH, MDA, SOD, IL-6, and IL-8 were examined by ELISA in the above groups. ^∗∗^*p* < 0.01 vs. control (ethanol); ^##^*p* < 0.01 vs. TNBS. (g) Protein levels of p-p65, p65NF-*κ*B, ICAM, GPX1, and p-I*κ*B*α* by Western blotting analysis. ^∗∗^*p* < 0.01 vs. control; ^#^*p* < 0.05, ^##^*p* < 0.01 vs. TNBS.

We established TNBS-induced colitis as a murine model of CD as our previous study (Figure 5(a)). First, we examined the SBP1 expression and found lower levels of SBP1 in the pre-miR-122 or si-SBP1-added TNBS group than the TNBS group (Figure 5(b)). Pre-miR-122 or si-SBP1 could obviously ameliorate the DAI and histology scores compared to the TNBS group (Figures 5(c)–5(e)). Meanwhile, we found lower levels of 8-OHdG, MDA, IL-6, and IL-8 and higher levels of GSH and SOD in the pre-miR-122 or si-SBP1-added TNBS group than the TNBS group (Figure 5(f)). At last, we also found that pre-miR-122 or si-SBP1 obviously inhibited the levels of p-p65, p65NF-*κ*B, and ICAM and enhanced the expressions of GPX1 and p-I*κ*B*α in vivo* (Figure 5(g)).

### 3.7. Inhibition of miR-122 Promoted H_2_O_2_-Induced Oxidative Stress *In Vitro*

**Figure 6:**
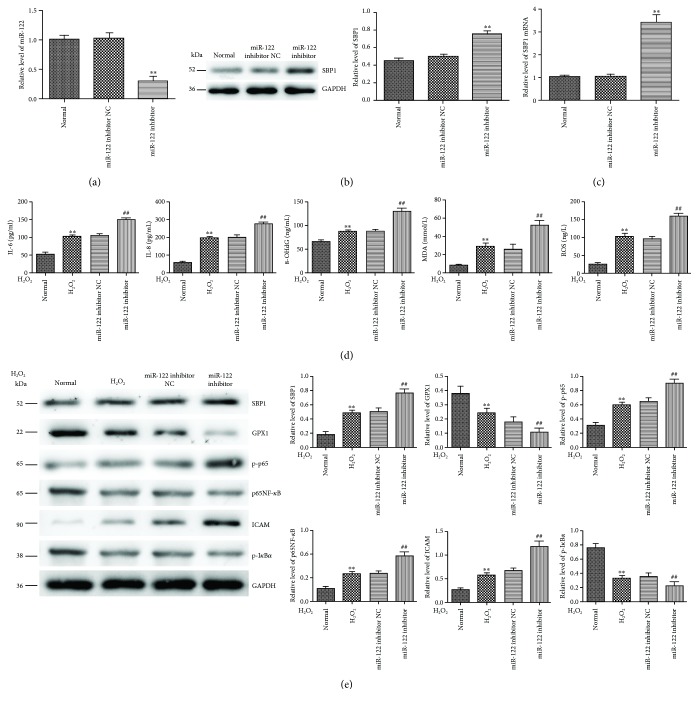
Inhibition of miR-122 promoted H_2_O_2_-induced oxidative stress *in vitro*. (a–c) miR-122, SBP1 mRNA, and protein level in HT-29 cells after transfection with the miR-122 inhibitor. ^∗∗^*p* < 0.01 vs. normal. (d) The protein levels of ROS, MDA, 8-OHdG, IL-6, and IL-8 were examined by ELISA in normal, H_2_O_2_, and miR-122 inhibitor-added H_2_O_2_ groups. ^∗∗^*p* < 0.01 vs. normal; ^##^*p* < 0.01 vs. miR-122 inhibitor NC. (e) Higher levels of p-p65, p65NF-*κ*B, and ICAM and lower levels of GPX1 and p-I*κ*B*α* were found in the miR-122 inhibitor-added H_2_O_2_ group than the H_2_O_2_ group. ^∗∗^*p* < 0.01 vs. normal, ^##^*p* < 0.01 vs. miR-122 inhibitor NC.

To further confirm the effects of miR-122 on H_2_O_2_-induced oxidative stress, we transfected the miR-122 inhibitor into HT-29 cells to evaluate its efficiency and found significant decreased expression of miR-122 compared to the control group (Figure 6(a)) followed by obviously increased expressions of SBP1 mRNA and protein (Figures 6(b) and 6(c)). These results further confirmed that SBP1 might be a direct target for miR-122. Then, cells pretransfected with the miR-122 inhibitor were stimulated with H_2_O_2_ (300 *μ*M) for the indicated times. Subsequently, we found a higher level of ROS, MDA, 8-OHdG, IL-6, and IL-8 in the H_2_O_2_ group than the normal group, while a higher level of them in the miR-122 group than the H_2_O_2_ group. Cotreatment of pre-miR-122 and si-SBP1 could intensify this phenomenon (Figure 6(d)). Subsequently, we examined the levels of ICAM and p65NF-*κ*B signaling related to oxidative stress and found significant activation of p65NF-*κ*B signaling and ICAM under H_2_O_2_ stimulation. Moreover, we found higher levels of p-p65, p65, and ICAM and lower levels of GPX1 and p-I*κ*B*α* in the miR-122 inhibitor-added H_2_O_2_ group than the H_2_O_2_ group (Figure 6(e)).

### 3.8. Inhibition of miR-122 Aggravated TNBS-Induced Colitis *In Vivo*

**Figure 7:**
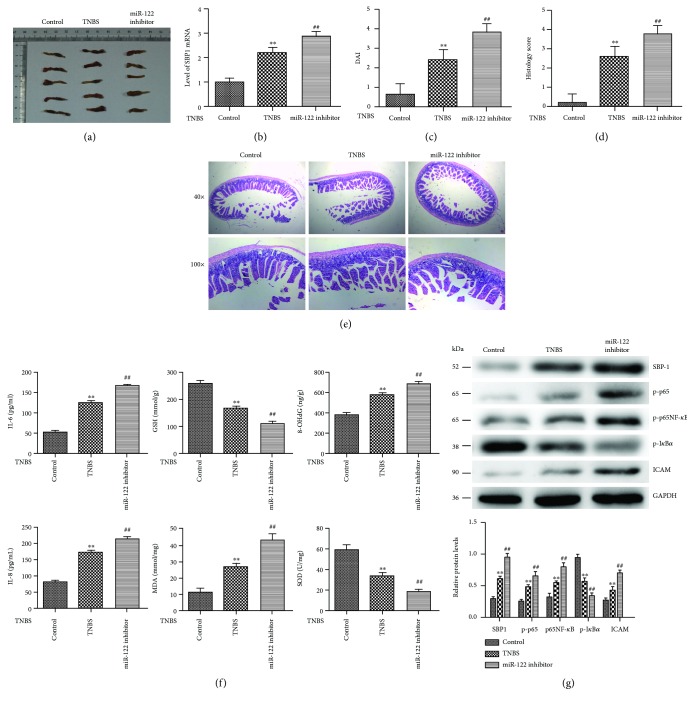
Inhibition of miR-122 aggravated TNBS-induced colitis *in vivo*. (a) The macroscopic appearance of the inflammatory colon in the control, TNBS, and miR-122 inhibitor-added TNBS groups. (b) qRT-PCR analysis of SBP1 in the above groups. (c–e) DAI and histology scores in the above groups. ^∗∗^*p* < 0.01 vs. control, ^##^*p* < 0.01 vs. TNBS. (f) The protein levels of 8-OHdG, GSH, MDA, SOD, IL-6, and IL-8 were examined by ELISA in the above groups. ^∗∗^*p* < 0.01 vs. control, ^##^*p* < 0.01 vs. TNBS. (g) Protein levels of p-p65, p65NF-*κ*B, ICAM, GPX1, and p-I*κ*B*α* by Western blotting analysis. ^∗∗^*p* < 0.01 vs. control, ^##^*p* < 0.01 vs. TNBS.

We established TNBS-induced colitis as a murine model of CD as our previous study (Figure 7(a)). First, we examined the SBP1 expression and found a higher level of SBP1 in the miR-122 inhibitor-added TNBS group than the TNBS group (Figure 7(b)). Inhibition of miR-122 could obviously aggravate the DAI and histology scores compared to the TNBS group (Figures 7(c)–7(e)). Meanwhile, we found high levels of 8-OHdG, MDA, IL-6, and IL-8 and lower levels of GSH and SOD in the miR-122 inhibitor-added TNBS group than the TNBS group (Figure 7(f)). At last, we also found that inhibition of miR-122 obviously promoted the levels of p-p65, p65NF-*κ*B, and ICAM and decreased the expression of GPX1 and p-I*κ*B*α in vivo* (Figure 7(g)).

### 3.9. Oxidative Stress Promoted DNA Methylation of miR-122

**Figure 8:**
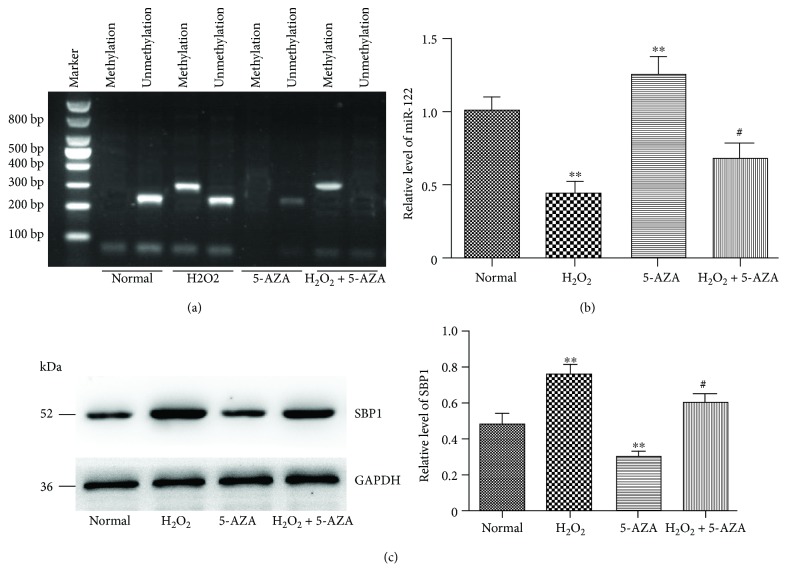
Oxidative stress promoted DNA methylation of miR-122. (a) CpG island methylation in the miR-122 promoter region under normal or H_2_O_2_ stimulation. (b, c) DNA demethylation with 5′-AZA increased miR-122 expression but decreased SBP1 expression. ^∗∗^*p* < 0.01 vs. normal; ^#^*p* < 0.05, ^##^*p* < 0.01 vs. H_2_O_2_.

In order to investigate whether DNA methylation regulated the expression of miR-122 under oxidative stress conditions, we compared the levels of DNA methylation. We found that the promoter region of miR-122 under oxidative stress conditions was overmethylated compared to the control group (Figure 8(a)). When 5′-AZA was present, the percentage of miR-122-methylated DNA sites was decreased significantly and the miR-122 level was increased (Figure 8(b)). Moreover, we also found that the level of SBP1 was decreased in the 5′-AZA group compared to the control group (Figure 8(c)).

## 4. Discussion

Crohn's disease (CD) is a chronic recurrent inflammatory disorder of the gastrointestinal tract with a high recurrence rate, canceration risk, and poor prognosis [30]. Although the treatment of CD has been improved to some extent, the etiology is still uncovered. Therefore, it is crucial to study the pathogenesis and develop new therapeutic targets of CD.

Several microRNAs have been identified as harmful or helpful factors in intestinal inflammation [31, 32] and are closely related to oxidative stress [33]. miR-122 was reported to regulate diverse biological functions in liver diseases [34] and improve the hepatic differentiation process in human adipose tissue-derived stem cells (hADSCs) [35]. However, the mechanisms of miR-122 in oxidative stress of CD have not been reported.

Oxidative stress has been relevant to some clinical features exhibited in CD such as fibrosis and tissue injury [6, 10, 36]. We employed H_2_O_2_ to induce the oxidative stress model in HT-29 cells and found a negative correlation between H_2_O_2_ concentration and the level of miR-122. It was obvious that the cell viability was markedly decreased under H_2_O_2_ stimulation.

SBP1, a selenium-binding protein, is known as an anticancer factor in several solid tumors and also participates in the regulation of oxidative stress [37, 38]. SBP1 had been reported to inhibit GPX1 activity to promote oxidative stress and reciprocally regulate hypoxia-inducible factor-1*α* (HIF-1*α*) in the oxidative stress model of hepatoma cells stimulated by H_2_O_2_ [39]. In our study, we found that the expression of SBP1 was increased in patients with active CD, TNBS-induced colitis model, and H_2_O_2_-induced HT-29 cells oxidative stress model. Then, we confirmed SBP1 as the target of miR-122 with the dual-luciferase reporter gene assay. Cotreatment of pre-miR-122 and si-SBP1 alleviated the activities on inflammatory response and oxidative stress compared to individual treatment of miR-122 or si-SBP1. Meanwhile, pre-miR-122 or si-SBP1 treatment alleviated the development of TNBS-induced CD; reduced the levels of 8-OHdG, MDA, IL-6, IL-8, and ICAM; and enhanced the expressions of GSH, GPX1, and SOD, which indicated an antioxidative stress effect and protection of intestinal mucosa in CD.

NF-*κ*B signaling, a master regulator of inflammation and also a sensor of oxidative stress, has a strategic position at the communication between oxidative stress and inflammation [29, 40]. We also detected the p65NF-*κ*B signaling *in vivo* and *in vitro*. Pre-miR-122 or si-SBP1 inhibited the levels of p-p65 and p65NF-*κ*B and enhanced the expression of p-I*κ*B*α* which is well known as an inhibitor of p65NF-*κ*B. Cotreatment of pre-miR-122 and si-SBP1 had better effects compared with the pre-miR-122 or si-SBP1 group as well. These results indicated that p65NF-*κ*B signaling played a vital role in miR-122/SBP1-related oxidative stress.

At last, we found overmethylation of the CpG rich region of miR-122 under H_2_O_2_ stimulation and a lower level of miR-122 and a higher level of SBP1 under H_2_O_2_ stimulation. However, when a DNA-demethylating agent (5′-AZA) was present, the level of miR-122 was increased, and SBP1 increased.

Taken together, overmethylation of miR-122 under oxidative stress induced a lower level of miR-122 and a higher level of SBP1 in colitis, which may suppress inflammatory response via p65NF-*κ*B signaling. Based on our findings, enhanced miR-122 expression indicates an effective and bright view for a promising therapeutic target in the clinical treatment of CD.

## Data Availability

The relative experimental methods used to support the findings of this study are included within the article. Our previously reported TNBS-induced colitis model is available at DOIs 10.1016/j.molimm.2015.07.032 and 10.1007/s10495-016-1301-y. Other raw data are included in the accessory listed as the format of GraphPad Prism.
