# Correction to “DNA Methylation of miR‐122 Aggravates Oxidative Stress in Colitis Targeting SELENBP1 Partially by p65NF‐κB Signaling”

**DOI:** 10.1155/omcl/9789842

**Published:** 2026-02-25

J. Bai, J. Yu, and J. Wang, et al., “DNA Methylation of miR‐122 Aggravates Oxidative Stress in Colitis Targeting SELENBP1 Partially by p65NF‐κB Signaling,” *Oxidative Medicine and Cellular Longevity* 2019 (2019): 5294105, https://doi.org/10.1155/2019/5294105.

In the article titled, “DNA Methylation of miR‐122 Aggravates Oxidative Stress in Colitis Targeting SELENBP1 Partially by p65NF‐κB Signaling,” there is an error in Figure 6, where a layout error was identified in the final draft of the article, involving repeated bands for p65NF‐κB and p‐IκBα, as well as inconsistent expression trends between p65NF‐κB and its corresponding bar chart on the right side. The correct Figure 6 is shown below.

Figure 6Inhibition of miR‐122 promoted H_2_O_2_‐induced oxidative stress in vitro. (a–c) miR‐122, SBP1 mRNA, and protein level in HT‐29 cells after transfection with the miR‐122 inhibitor.  ^∗∗^ *p* < 0.01 vs. normal. (d) The protein levels of ROS, MDA, 8‐OHdG, IL‐6, and IL‐8 were examined by ELISA in normal, H_2_O_2_, and miR‐122 inhibitor‐added H_2_O_2_ groups.  ^∗∗^ *p* < 0.01 vs. normal; ^##^ *p* < 0.01 vs. miR‐122 inhibitor NC. (e) Higher levels of p‐p65, p65NF‐κB, and ICAM and lower levels of GPX1 and p‐IκBα were found in the miR‐122 inhibitor‐added H_2_O_2_ group than the H_2_O_2_ group.  ^∗∗^ *p* < 0.01 vs. normal, ^##^ *p* < 0.01 vs. miR‐122 inhibitor NC.

**Figure:**
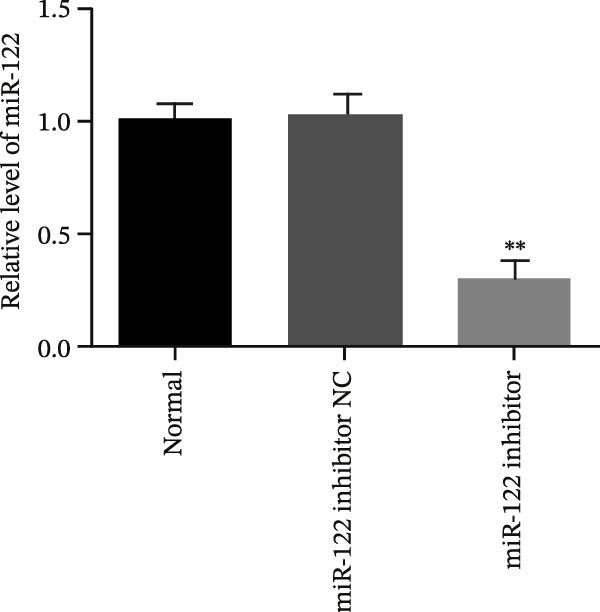
(a)

**Figure:**
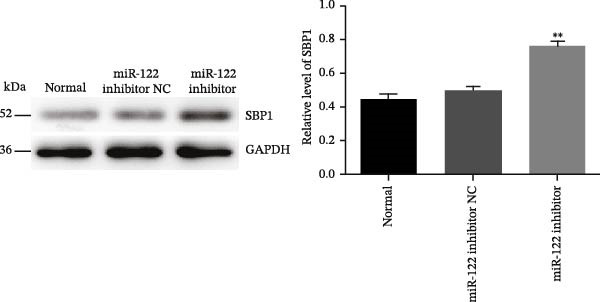
(b)

**Figure:**
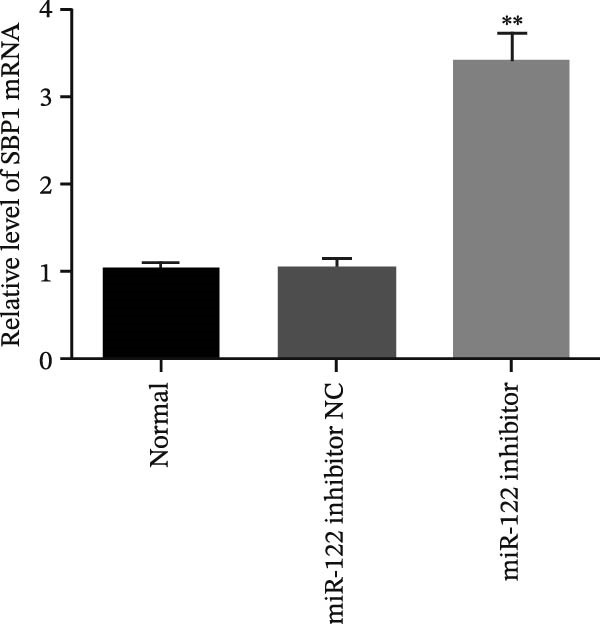
(c)

**Figure:**
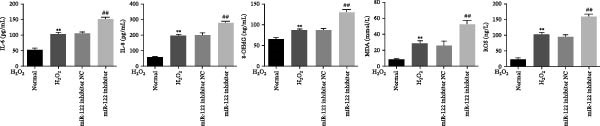
(d)

**Figure:**
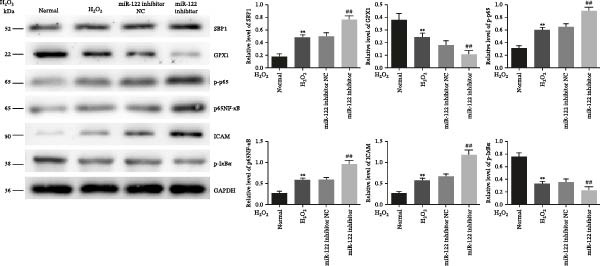
(e)

We apologize for this error.

